# Palliative outpatient parenteral antibiotic therapy: a review of 5 years of patient data

**DOI:** 10.1093/jacamr/dlaa052

**Published:** 2020-08-06

**Authors:** Elizabeth Hart, Sue Snape, Ross Thomson

**Affiliations:** 1 Nottingham University Hospitals, Hucknall Road, Nottingham NG5 1PB, UK; 2 Division of Primary Care, University of Nottingham, University Park, Nottingham NG7 2RD, UK

## Abstract

**Objectives:**

A review of patients requiring lifelong antibiotics to control, rather than cure, infection was performed [‘palliative outpatient parenteral antibiotic therapy (OPAT)’]. This was to evaluate emerging themes and complications. The aim was to aid in the management of such patients.

**Methods:**

A retrospective review of the OPAT database over 5 years (2013–17) was performed. Of the 1438 patients, 9 were deemed to have received palliative OPAT.

**Results:**

The palliative cohort represented 0.6% of the total number of patients on OPAT and 8.6% of the bed days saved. Patients fell into two main groups: those with multiple comorbidities that precluded surgical management and those with a terminal condition. Both groups received IV antibiotics with no clear endpoint. The themes to emerge were: patients often had multiple comorbidities with a high operative risk to control the source of infection; a trial of no or oral antibiotics led to resurgence of the infection; vascular patients appeared to tolerate long-term antibiotics well; and conversely, antibiotic side effects were a significant issue in others. Patients with incurable cancer and a coincident infection can be given additional quality of life with the judicious use of appropriate therapy.

**Conclusions:**

There are significant issues surrounding antimicrobial stewardship in the palliative OPAT group that should be considered. Excellent communication is required to deal with these often very complicated patients. There are considerable gains to be made both for patients and the number of bed days saved. The small number of patients accounted for a disproportionate number of bed days saved.

## Introduction

Outpatient parenteral antibiotic therapy (OPAT) is a method for delivering IV antimicrobials in the community or outpatient setting as an alternative to inpatient care. Since first described in 1974 for the management of paediatric patients with cystic fibrosis,^1^ OPAT has demonstrated improvements in the quality of life for patients for whom administration of IV antibiotics is their primary reason for inpatient hospital care. It has also reduced the associated costs of prolonged hospital admissions.^2–5^

In the UK, patient acceptance and clinical experience with OPAT are increasing, becoming part of routine recommendations for many chronic, subacute and acute infections. Principles and practice of OPAT recommend that infections treated should have a low probability of progression with a predictable response to therapy and the outcome for patients should be a more rapid return to normality, work or education.^6^ A small number of patients do not meet these recommendations but still require lifelong OPAT as part of palliative care. Although few in number, these patients have required a considerable investment of time and resources with regard to planning their antibiotic combinations and managing their conditions.

Recent good practice recommendations have acknowledged palliation as a treatment aim.^7^ This paper aims to share our experience in meeting the needs of this particular patient group.

## Methods

Our working definition of palliative OPAT refers to patients who require lifelong antibiotics to control rather than cure any infection. They are discharged from hospital with the intention that the antibiotics continue lifelong, although this has not always been possible. It is understood that if the antibiotics are stopped then this will lead to resurgence of the underlying infection.

The Nottingham University Hospitals NHS Trust OPAT service was established in 2011 in the regional Infectious Diseases Unit, based in a large teaching hospital. In the time frame reviewed, the service followed the Good Practice Recommendations 2012 standard of care with a multidisciplinary team approach comprising microbiologists, infectious diseases physicians, a specialist nursing team and an antimicrobial pharmacist.^8^ The service uses two models of antibiotic delivery. The first takes place at an outpatient ‘infusion centre’ in the hospital and the second is patient/carer or nurse administration in the patient’s home. The latter requires weekly visits to the outpatient clinic as per UK national good practice recommendations. There is 24 h access for admission, as necessary, for all OPAT patients.

The database was reviewed retrospectively for those patients who had received prolonged antibiotics and those with a diagnosis of palliative conditions (see Tables 1 and 2). Over the 5 year period (2013–17), nine patients were identified who commenced OPAT with no expectation of a curative outcome. Patients who received repeated cycles of OPAT for the same condition (e.g. bronchiectasis) were excluded. During this period the OPAT database changed and, if it was not possible to clarify the length of time antibiotics were given, the clinical letters were reviewed to discover the appropriate data.

**Table 1.** Patients who had infection with an inoperable source

Patient number	Age at start (years)	Pathology	Microbiology	Antibiotics (IV) and method of administration	Time on OPAT	Comments	Outcome
1	81	Infected aortic graft, no procedure possible due to frailty, responded to ertapenem	*Escherichia coli*	Ertapenem 1 g once a day to 500 mg once a day Self-administration	38 months	Death unrelated to infection after prolonged decline Dose modification as renal function declined due to comorbidities	deceased
2	65	Infected aortic graft, no curative procedure possible	*E. coli*, *Enterococcus faecium* (VRE)	Tigecycline 50 mg twice a day Self-administered	32 months continuing	Has required occasional insertion of drain into infected mass, decision made clinically and as CRP increases	alive
3	81	Infected aortic graft, no curative procedure possible	*E. coli*	Ertapenem 1 g once a day OPAT clinic	11 months	Relapsed on oral ciprofloxacin, requiring drainage in response to clinical symptoms and rising CRP	alive
4	78	Inoperable pelvic abscess (too frail to survive surgery due to multiple comorbidities)	Coliform, *Enterococcus*	Ceftriaxone 2 g once a day (with oral metronidazole) Followed by daptomycin 500 mg once a day and ertapenem 1 g once a day Nurse delivered	7 weeks	Changed treatment due to severe nausea, vomiting and diarrhoea, then stopped altogether as unable to tolerate, multiple discussions with regard to implications of stopping requiring MDT involvement: remained alive 4 weeks after stopping treatment	deceased
5	79	Inoperable pelvic abscess (too frail to survive surgery due to multiple comorbidities)	*E. coli*, anaerobes, *Actinomyces*, *Enterococcus gallinarum*, *Enterobacter*	Meropenem 500 mg twice a day Nurse delivered	8 weeks	Readmitted due to physical decline and inability to cope and decision made to stop antibiotics as unable to monitor and concern that risks were outweighing benefits	deceased
6	75	Infected EVAR graft	Nil grown but septic when off treatment	Daptomycin 400 mg once a day Nurse delivered	4.5 months	Failed initial finite IV and then severe side effects with oral antibiotics before restarting IV	deceased
7	60	Discitis, infected clot in immovable IVC filter, vascular malformation and osteomyelitis right leg	MRSA	Vancomycin 500 mg to 1 g twice a day dependent on twice-weekly levels Nurse delivered	17 months	MDR MRSA meant that vancomycin was the only viable option; death due to intracerebral haemorrhage	deceased

CRP, C-reactive protein; MDT, multidisciplinary team; EVAR, endovascular aneurysm repair.

**Table 2.** Patients with a terminal condition where OPAT was used to treat coincident infection

Patient number	Age at start (years)	Pathology	Microbiology	Antibiotics (IV) and method of administration	Time on OPAT	Comments	Outcome
8	70	Infected TKR, further procedures unsafe due to resistant AML with persistent neutrophil count of zero	CoNS	Teicoplanin 800 mg once a day initially then daptomycin 500 mg once a day due to resistance Family delivered	7 months	Died due to underlying illness	deceased
9	60	Sepsis on a background of metastatic colorectal cancer	MSSA	Ceftriaxone 2 g once a day then 24 h flucloxacillin due to relapse of sepsis secondary to infected portacath Nurse delivered	19 days in total	Two admissions; although short period on OPAT, this allowed him time at home with his family that he would not otherwise have had Flucloxacillin lower MIC compared with third- generation cephalosporins for MSSA	deceased

TKR, total knee replacement.

### Ethics

This work was deemed to be a service evaluation of palliative patients to help inform future care of this cohort and therefore, in line with NHS Health Research Authority guidelines, ethics approval was not required.^9^

## Results

During a 5 year period, we identified nine patients who met our definition of palliative OPAT. The number of bed days saved for this small number of patients was 3406, reflecting the length of time that patients can tolerate this form of management. The total number of OPAT patients during this period was 1438. The palliative cohort represented 0.6% of the total number of patients and 8.6% of the bed days saved.

Informal qualitative review of patient and infection characteristics identified two distinct groups and six recurrent themes.

Two groups of patients were identified. In the first group (Table 1) OPAT was used for the primary condition where there were no oral options or definitive surgical treatments possible. This group was made up of patients with vascular graft infections and pelvic abscesses. The second group (Table 2) consisted of patients with a terminal condition where OPAT was used to treat coincident infections. Common themes to emerge on review of all patients were: (i) the presence of multiple comorbidities; (ii) the underlying condition was inoperable, often due to high anaesthetic risk; (iii) a trial of IV antibiotics with or without oral antibiotics (if resistance suggested that this was an option) led to relapse of the infection; (iv) those who experienced multiple side effects with antibiotics struggled with prolonged courses; (v) vascular patients with deep-seated infections seemed to tolerate long-term antibiotics well; and (vi) patients with incurable cancer and a coincident infection can be given additional quality of life with the judicious use of appropriate therapy.

### Patient case studies

We present four case studies that are representative of the patients who have commenced long-term OPAT to illustrate the issues and learning points.

#### Patient 1

An 81-year-old man had an elective repair of an abdominal aortic aneurysm in 2008. The procedure was complicated by infection and bleeding. He relapsed on oral antibiotics multiple times and, in 2013, was attending the vascular ward on a daily basis for IV antibiotics. He was referred to OPAT. He learnt to administer his own antibiotics using prefilled devices and was able to go on holiday. He died some years later of unrelated causes. OPAT allowed him to regain his independence and improved the quality of his life. This was our first patient on palliative OPAT and this relative success story made us more receptive to other cases.

#### Patient 4

A 78-year-old woman had a deep pelvic collection of uncertain origin that was inoperable due to her comorbidities. She had had six inpatient admissions over the previous 6 months for sepsis. She had been treated with short courses of IV antibiotics and had relapsed on oral regimens. After much discussion she was discharged on OPAT, on the understanding that this would be lifelong and with the intention of allowing her to remain at home. She managed to complete 8 weeks of OPAT before significant antibiotic-related side effects of nausea, vomiting and diarrhoea precluded continuation. Side effects severely impacted on her quality of life. Cessation of treatment required extensive conversations with the patient, the GP and the family to ensure that all understood the implications of stopping antibiotics and the impact that the antibiotics were having on the patient’s quality of life. OPAT was intended to provide better quality of life and prevent hospital admission. In addition, there was difficulty gaining blood on a weekly basis for monitoring. This led to concerns regarding the balance between side effects and the actual benefit of treatment. OPAT failure led to clear end-of-life discussions that involved the patient and her family and prevented further hospital admissions. The balance between suppression of infection and manageable side effects is important to acknowledge and sometimes the right decision is to stop treatment.

#### Patient 7

A 60-year-old man presented with a haemorrhagic cerebral infarct. During the course of his hospital admission he developed pulmonary emboli requiring the insertion of an inferior vena cava (IVC) filter. He subsequently developed MRSA sepsis secondary to an infected wound on his foot. He was readmitted with discitis felt to be secondary to MRSA. The IVC filter could not be removed. After a full course of treatment, he relapsed with concern that the clot in his IVC filter was chronically infected. The MRSA was multi-resistant and attempts at suppression with the limited choice of oral antibiotics failed due to side effects. OPAT allowed him to be discharged to spend the rest of his life nearer his family and out of hospital. The challenges of managing a multi-resistant bacterial infection in a community setting were a learning process for all involved.

#### Patient 8

A 70-year-old man was diagnosed with AML in July 2017. This proved refractory to treatment and he was maintained on regular blood and platelet transfusions. He had a neutrophil count of zero despite regular granulocyte colony stimulating factor (GCSF). In September 2017, he developed a CoNS infection in a previous total knee replacement. Surgery was felt to be too risky due to his profound pancytopenia and he was commenced on teicoplanin. In November 2017, his knee became more painful and swollen. Repeat aspiration showed that the bacteria had become resistant to teicoplanin. He was changed to daptomycin. He remained as an outpatient, attending haematology regularly for appropriate transfusions until his death in April 2018. OPAT kept him out of hospital during the last months of his life. It reduced the amount of pain he experienced and kept him mobile. This man had a very engaged family who appreciated that the antibiotics were controlling rather than curing the infection due to the low neutrophil count.

## Discussion

We have identified nine patients seen by our service over a 5 year period in whom the term palliative OPAT is appropriate. We have presented four case studies that are representative of the patients who have commenced palliative OPAT to illustrate issues and learning points. In the small number of patients we have seen, a range of themes emerged, as detailed earlier. Of note is the percentage of bed days saved (8.6%) compared with the percentage of palliative patients represented on the OPAT database (0.6%).

### Improved quality of life

The use of OPAT in patients with little or no expectation of a curative outcome is an uncommon but important component of the care our unit provides. The use of OPAT for palliation as opposed to cure is analogous to other end-of-life care arrangements for cancer and long-term conditions. The purpose of palliative care is to improve a patient’s subjective well-being by holistic, multidisciplinary and family- as well as patient-centred interventions.^10^ Home-based palliative care has been found to produce greater improvements in independence and security, while safeguarding patients’ entire life situation and increased quality of life when compared with that of patients admitted to hospital.^11^^,^^12^ In our experience, we have found that it has provided freedom from the requirements of hospital admission and provides patients and their families time in their own environment to achieve as much quality of life as possible. While the majority of patients receive daily nursing input to administer the antibiotics, for some patients it has proven possible to foster greater independence by training them to deliver their own antibiotic therapy. With organization, we have also arranged for a patient on lifelong OPAT to travel and have their first UK holiday in years.

### Communication

Communication is a key element of high quality in palliative care and the scope and timing of discussions between patient, family, clinicians and the discharging team are particularly important.^13^ Appropriate and timely discussion with the patient and the family should start with the discharging team before OPAT is started. In general, patients have expressed a preference for discussions around end-of-life care and ‘do not resuscitate’ orders to occur in a private, quiet place with the full attention of a physician who is able to communicate their diagnosis, prognosis and treatment options clearly. Patients appreciate the time to ask questions and to be given information about how the diagnosis and treatment will affect their life and these discussions should occur prior to discharge.^14^^,^^15^ This discussion can then lead on to ensuring patients have a realistic expectation of what OPAT can offer and what it can and cannot achieve. In the future, we aim to increase staff training to increase staff confidence in discussing palliative and end-of-life care.

Palliative OPAT in our setting has been most successful when there has been excellent liaison with the parent specialities who need to understand the limitations of OPAT with regard to side effects, logistics and the fact that a cure is not going to be possible. Effective communication between healthcare providers, the patient and the family is integral to optimal care.^16^ The patient’s GP and potentially palliative care teams should also be involved, as necessary, and the intricacies of this interaction should be resolved, ideally before discharge. There should be clear documentation of the aims of the treatment and a transparent declaration of its limitations.

### Monitoring on OPAT

In-depth conversations with the patient and family need to address the pros and cons of prolonged use of antibiotics and plans for monitoring must be clearly laid out. Patient 4 in Table 1 suffered from severe side effects from antibiotics that required her to stop antibiotics after much discussion. Patient 5 could not be monitored weekly for side effects of antibiotics. A decision was made that we could not continue with OPAT as there were concerns that the antibiotics would lead to her premature death. Both of these patients had deep pelvic abscesses and comorbidities prevented surgical treatment. Conversely, Patient 1 had been on OPAT for so long and had remained stable that monitoring took place every 2 weeks.

### Updated Good Practice Recommendations

When these data were collected, the Good Practice Recommendations 2012^8^ classed any death whilst on OPAT as a failure of treatment and OPAT care. The recently published Good Practice Recommendations 2019^7^ acknowledge the need for individualized treatment aims and categorizes palliation as a treatment aim. In these circumstances, death is an expected outcome rather than a failure and enabling a patient to stay at home during their last days undoubtedly improves quality of life.

### Antimicrobial stewardship

There are antimicrobial stewardship issues with regard to the prolonged use of antibiotics, namely those of antimicrobial resistance.

If antimicrobial resistance continues to increase, palliative OPAT may become increasingly relevant as effective oral options may not be available. Gram-negative infections are already a source of major concern.^17^ In the patients described above we have not managed untreatable infections but two of the cohort did develop organisms that became more resistant and required modification of their antibiotics, whereas another patient had no oral options available. This remains a concern for future practice.

In addition, the role of antibiotics with a prolonged half-life can play a pragmatic role. Teicoplanin has been used on a thrice-weekly basis at other centres^18^ and this could lend itself to a long course of treatment, as in osteomyelitis.^19^ Dalbavancin is licensed for a maximum of two doses at weekly intervals. Off licence it has been used with success on a longer-term basis.^20^ A reduced number of doses of antibiotic per week lends itself to outpatient treatment. At present, data on efficacy and safety are limited.

An alternative method of reducing the frequency of administration is 24 h infusion of antibiotics. In our unit, this mode of delivery is slowly increasing. Stability data are available for piperacillin/tazobactam^21^ and flucloxacillin.^22^ We used this method of delivery for flucloxacillin in a patient whose deep-seated MSSA infection relapsed as soon as his initial treatment had finished. It was not possible to remove the source of the infection. As the MIC of flucloxacillin is considerably lower than that of the third-generation cephalosporins for MSSA, this was felt to be a safer option.^23^

### Future plans

We believe that palliative OPAT is a relevant use of OPAT resources for the small number of patients to whom it is applicable. It can improve quality of life as well as keeping patients at home when resources are limited. We hope to encourage the use of OPAT in such situations within the hospital environment so that more specialities are aware of the potential possibilities and also have an understanding of the logistics involved in appropriate discharge. It is essential that all areas of patient care have been adequately addressed. We aim to focus on patient feedback to see how our service could improve. We are also exploring increased liaison with our palliative care teams to ensure that clear and swift pathways of communication are in place to ensure holistic patient care is delivered.

### Conclusions

Palliative OPAT can be successful and lead to positive outcomes for patients and their families. It should be planned with sympathetic, frank and pragmatic conversations with all concerned. Our experience of this complex group of patients is that it can achieve considerable gains with regard to quality of life for patients and their families.

## Supplementary Material

dlaa052_Supplementary_Data
