# Supplementary material for: Palliative outpatient parenteral antibiotic therapy: a review of 5 years of patient data
Source: JAC Antimicrob Resist. 2020 Aug 6;2(3):dlaa052. doi: 10.1093/jacamr/dlaa052 (PMC8210122; doi:10.1093/jacamr/dlaa052)
Supplement: dlaa052_Supplementary_Data [file dlaa052_supplementary_data.docx]

**Reviewer report 1 (R0 – review of original submission)**

**PLEASE NOTE: JAC-AMR aims to publish your anonymous peer review comments with the final article for transparency. Please indicate in the box below whether you give your consent for us to publish your peer review comments for this article.**

Yes

**Synopsis**

**1. If the article contains original data, does the synopsis adequately reflect the manuscript as a whole, with the most important aspects described (of the introduction, methods and key results, and a balanced conclusion)? If NO, what is required to improve the synopsis? Please use the box below.**

Yes

Can you edit synopsis in light of required revisions, in particular about the various themes identified (see below)

**Introduction**

**2. Does the introduction provide the necessary background information for readers to understand the article? (e.g. does it describe the problem, current state of knowledge, etc.) If NO, what is required to improve it? Please use the box below**

Yes

**3. Is the research hypothesis and/or aims/objectives of the study clearly stated in the introduction? If NO, what is required to improve it? Please use the box below**

Yes

**4. Is there superfluous/unnecessary text in the introduction? (Only text that is required for readers to understand why the study has been done should be included). If YES, what text is superfluous and should be removed? Please use the box below**

No

Considering the answers above and any other comments/advice you may have, please indicate what is required to improve the Introduction:

L29 Comma instead of full-stop needed after "..cystic fibrosis"

L37-39 The sentence "During the five years reviewed we have been presented with a small number of patients who have not met the above recommendations but have required lifelong antibiotic treatment." requires rewording. Perhaps to: "We are aware that a small number of patients do not meet the above recommendations but require lifelong OPAT as part of palliative care." or similar...

L42-44 Please change to the following or similar: "Recent good practice recommendations have acknowledged palliation as a treatment aim in its own right. This paper aims to share our experience in meeting the needs of this particular patient group."

L45-57 I would put all this bit within methods please

**Methods**

**5. Were the methods used suitably robust and appropriate for the research question(s) and/or the aims/objectives of the study? If NO, the article should be rejected, but please advise the authors accordingly in the free-text box below and complete the review in full**

Yes

**6. Are the methods described in enough detail so the reader can understand what was done and interpret the results and conclusions appropriately?**

Yes

**7. Are the methods described in language that a reader who does not specialize in the area of the study/methods used will understand? For example, are the statistical approaches used described in language that non-statistician healthcare professionals will understand? If NO, please indicate what is required to improve the language in the methods section in the box below**

Yes

Considering the above and any other comments/advice you may have, please indicate other aspects of the methods section that could be improved in the free-text box below)

See above comments in Introduction regarding moving of text

**Results**

**8. Given the methods used, are results described accurately, in enough detail and in an appropriate format to adequately inform the discussion section and the authors/readers conclusions? If NO, please indicate what is required to improve the results section in the box below**

No

**9. Based on what is described in the methods section, are data/results (that are likely to be available to the authors) missing from the current draft that you think should be presented to improve the manuscript?**

Yes

**10. Are Tables/Figures clear, required and do they add/complement the results text (they should not simply repeat what is in the text or vice versa)? If NO, please indicate what is required to improve the Tables/Figures in the box below**

Yes

Considering the above and any other comments/advice you may have, please indicate what is required to improve the results section:

Could you start "Results" with a statement such as "During five years, we identified 9 patients (include % of total OPAT patients treated in that time) who were defined by our definition as palliative OPAT."

L71 "Common themes to emerge on review of all patients are:" - two comments 1) "...were:" is probably more appropriate than "...are:" - in synopsis too... AND 2) did you use a formal method of qualitative analysis to identify the emerging themes or was this more of an informal type of analysis - please state this in the text so readers understand - thanks. See also comment about emerging themes under Discussion section

L96-98 - this sentence needs some punctuation and possibly rewording I think - perhaps: "Given the side effects, quality of life was not good on discharge. The failure of OPAT however, led to clear end of life discussions that involved the patient and her family and prevented further hospital admissions." or similar...

Comment: Palliative OPAT obviously leads to a high number of bed days saved/DDDs/days of OPAT for a relatively small nos of patients - it is potentially important for OPAT services to be aware of this and audit such data accordingly - could you present that data in the manuscript please for the patients presented as I think readers will be interested to see the discrepancy between the number of patients on palliative OPAT (versus overall OPAT) and the % of days of OPAT taken up by this relatively small number of patients - Thanks.

Comment: You mention side-effects both in text and table but don't provide any detail - could you outline the nature of those side effects please?

Table 1:
1) Not sure if this is just the conversion on the system, but in Column 3 "Pathology" can you ensure consistency in use of capitals at beginning of sentence - also for Antibiotic column too (e.g. vancomycin) and for Table 2 too - thanks.
2) Column 4 "Micro" - please spell in full - thanks
3) Row 3: Full stop after E of E coli please
4) Can you provide the exact dose regimens (but especially for patients 2 and 5) of the regimens used and how delivered (daily outpatient, 24h infusion, thrice weekly, etc, etc...)

Table 2:
Patient 8 - In col 3 can you change to "... neutrophil count..."

**Discussion**

**11. Are the discussion and any conclusion(s) reasonable given the stated hypothesis/aims/objectives and results? If NO, please indicate how to improve the discussion in the box below**

Yes

**12. Is there superfluous/unnecessary text in the discussion? (Only text that directly relates to the results and understanding thereof in the context of existing published literature and the limitations of the study should be included.) If YES, please indicate what should be removed to improve the discussion in the box below**

No

**13. Is the Limitations sub-section adequate and reflective of the actual limitations of the study? ? If NO, please indicate how to improve the Limitations section in the box below**

Not applicable

Considering the above and any other comments/advice you may have, please indicate how to improve the discussion section:

L129-131: These themes are different to the themes under the "Results" section - I suggest you amalgamate all the emerging themes and present them in the same section (probably Results) or in a table referenced from the text (don't repeat themes in text and table please if you decide on a table) - Thanks.

**General**

**14. Is referencing throughout the manuscript satisfactory? (for instance are there statements for which you believe a supporting reference should be cited?) If NO, please indicate how to improve referencing in the box below**

Yes

**15. Is the language used (i.e. spelling, grammar, etc.) of high enough quality to be published as is? If NO, please indicate how to improve the language within the article in the box below**

Yes

**16. Are ethics/other approvals appropriate/adequate and satisfactorily described? ? If NO, please indicate how to improve this in the box below**

No

Considering the above and any other comments/advice you may have, please advise the authors how to improve these aspects of the article.

L161-62: Change to Effective communication between health care providers, the patient and the family is integral to optimal care."

L167: Change to "Detailed conversations with the patient and family need to address......"

L183-88: You may also want to mention however that if antibiotic resistance continues to increase, especially in Gram-negatives, that palliative OPAT may in fact become more common and necessary because of the lack of availability of oral options

Comment: it would be nice for you to discuss something about the role of antibiotics with a prolonged half-life in palliation - particularly for Gram+ infections - (e.g. that can be given twice weekly (teicoplanin) or less (dalbavancin)) - and how more research is required with respect to dosing regimens etc...

Approvals: Can you clarify and state in your manuscript somewhere what approvals were sought (e.g. service evaluation)

**Opinion and review articles only**

**17. Please advise the authors in the box below on how to improve their opinion or review article:**

NA

|  **Recommendation** | |
| --- | --- |
|  | Accept (as is with no revisions) |
|  | Minor revision (with anticipation of subsequent acceptance if revisions are adequate) |
|  | Major Revision |
|  | Reject |

| **Would you be willing to review a revision of this manuscript?** | |
| --- | --- |
|  | Yes |
|  | No |

**Comments**

 Comments to the Author

If you have any additional comments to the authors about any aspect of the manuscript not covered in the structured review above, please place these in the box below. If you have no additional comments, please say “No additional comments” in box.

See my comments within structured review please - thanks
